# Both Stationary and Dynamic Functional Interhemispheric Connectivity Are Strongly Associated With Performance on Cognitive Tests in Multiple Sclerosis

**DOI:** 10.3389/fneur.2020.00407

**Published:** 2020-06-04

**Authors:** Sue-Jin Lin, Shannon Kolind, Aiping Liu, Katrina McMullen, Irene Vavasour, Z. Jane Wang, Anthony Traboulsee, Martin J. McKeown

**Affiliations:** ^1^Graduate Program in Neuroscience, University of British Columbia, Vancouver, BC, Canada; ^2^Division of Neurology, Department of Medicine, UBC Hospital, University of British Columbia, Vancouver, BC, Canada; ^3^Department of Electrical and Computer Engineering Program, University of British Columbia, Vancouver, BC, Canada; ^4^Department of Radiology, Faculty of Medicine, University of British Columbia, Vancouver, BC, Canada

**Keywords:** static functional connectivity, dynamic functional connectivity, multiple sclerosis, symbol digit modalities test, paced auditory serial addition test, resting state fMRI

## Abstract

Although functional connectivity has been extensively studied in MS, robust estimates of both stationary (static connectivity at the time) and dynamic (connectivity variation across time) functional connectivity has not been commonly evaluated and neither has its association to cognition. In this study, we focused on interhemispheric connections as previous research has shown links between anatomical homologous connections and cognition. We examined functional interhemispheric connectivity (IC) in MS during resting-state functional MRI using both stationary and dynamic strategies and related connectivity measures to processing speed performance. Twenty-five patients with relapsing-remitting MS and 41 controls were recruited. Stationary functional IC was assessed between homologous Regions of Interest (ROIs) using correlation. For dynamic IC, a sliding window approach was used to quantify changes between homologous ROIs across time. We related IC measures to cognitive performance with correlation and regression. Compared to control subjects, MS demonstrated increased IC across homologous regions, which accurately predicted performance on the symbol digit modalities test (SDMT) (*R*^2^ = 0.96) and paced auditory serial addition test (PASAT) (*R*^2^ = 0.59). Dynamic measures were not different between the 2 groups, but dynamic IC was related to PASAT scores. The associations between stationary/dynamic connectivity and cognitive tests demonstrated that different aspects of functional IC were associated with cognitive processes. Processing speed measured in SDMT was associated with static interhemispheric connections and better PASAT performance, which requires working memory, sustain attention, and processing speed, was more related to rigid IC, underlining the neurophysiological mechanism of cognition in MS.

## Introduction

As multiple sclerosis (MS) is characterized by discrete lesions visible on standard T2-weighted MRI images, there has been traditional emphasis on establishing the clinical–pathologic correlation between lesion location and symptomatology, including cognitive functioning (1, 2). Resting-state functional MRI (rs-fMRI) has been used to explore the more spatially distributed aspects of neural correlates of cognitive dysfunction, which can affect up to 70% of patients with MS and contribute to an impaired quality of life (3). Among the myriad of cognitive deficits seen in MS, information processing speed is especially important, as it is commonly impaired and important for performance in many cognitive domains (4). Linking processing speed deficits and functional connectivity has been a trend to further understand cognitive disease sequelae.

The corpus callosum (CC) facilitates interhemispheric connections in humans and lesions in the CC damage such connections (5, 6), resulting in numerous clinical deficits (7–11). For example, structural neuroimaging suggests that white matter lesions involving callosal connections predict disability in MS (7). Structural changes in the CC such as callosal atrophy is related to lower processing speed abilities in MS, as measured by the Symbol Digit Modalities Test (SDMT) at both baseline and follow-up (12). Abnormal callosal fractional anisotropy is correlated with impairment in several behavioral measures such as functional composite scores, processing speed, and performance of upper extremity function (13).

In addition to altered structural interhemispheric connectivity (IC), studies of functional IC provide insights into deficits seen in MS. Functional connectivity measures of homologous regions derived by electroencephalography are associated with CC atrophy in MS (14). Although no correlations between functional measures and behavior are reported, this study reveals the association between structural changes in CC and functional IC. Decreased magnetoencephalography synchronization between the two hemispheres has also been found in MS, suggesting that weakened functional IC could be a biomarker for cognitive impairment (15). Homotopic functional connectivity has been shown to be altered in visual, somatosensory, motor, and sensory processing regions in MS (16), and such alterations are correlated with microstructural damages.

The two most commonly used tests for detecting cognitive deficits in MS are the SDMT and the Paced Auditory Serial Addition Test (PASAT). PASAT was originally developed to test the speed of information processing; however, owing to its complexity, the performance also requires a great deal of sustained and divided attention, working memory, and processing speed, so it is sensitive to deficits (17). SDMT, on the other hand, also measures processing speed ability, but the task does not involve complicated components across multiple cognitive domains and thus has been frequently used as a neuropsychological screening test for processing speed (18). Both tests have been recommended as the standardized tools for cognitive assessment in MS (19) and have been adapted to task-driven fMRI research. Studies have investigated effective functional connectivity among the test-specific regions and shown that information flow between two hemispheres is required for both SDMT and PASAT performance (20, 21).

There is a growing interest in *dynamic* functional connectivity (dFC), that is, how functional connectivity fluctuates over time, which may be a strategy to coordinate information flow across time and adapt to stimuli (22, 23). Traditional analyses estimate mean functional connectivity over the entire time course without considering the changes of connectivity strength over time. However, the brain is obviously dynamic, and thus, connectivity patterns may change constantly (24). A standard approach for calculating dFC is to use a sliding window approach to calculate connectivity within each window with or without overlap between windows (24, 25), revealing the variations in connectivity over time. Connectivity fluctuations are associated with performance in several cognitive domains such as attention, memory, executive function, and cognitive flexibility (26–32). A few studies have recently demonstrated that dFC is associated with cognitive performance in MS. Performance on processing speed, working memory, and executive function tests has been associated with higher variation of both global and specific (e.g., interhemispheric connections) resting-state functional connectivity (i.e., higher dFC) in MS (33). Another study has reported that greater variability of connectivity within the default mode network is related to better performance in a processing speed task (34).

In this study, we proposed that IC is of interest in MS and examined both *stationary* and *dynamic* aspects of IC and specifically related them to processing speed performance. First, we evaluated stationary functional IC in MS during resting-state fMRI. In addition, we applied a sliding window approach with calculations on connectivity differences to quantify the variation of connectivity in IC. We then applied regression and correlation analyses to investigate the relationships between stationary/dynamic functional IC, clinical data, and performance on processing speed tests. The overarching goal was to investigate whether functional stationary and dynamic IC are altered in MS and whether the IC measures are related to the subjects' processing speed performance in early stages of the disease.

## Materials and Methods

### Subjects

We recruited 25 patients with relapsing-remitting MS (mean age ± SD = 37.2 ± 9.5; 10 male and 15 female) in a Phase III randomized, double-blind, double-dummy trial to evaluate the efficacy and safety of ocrelizumab vs. interferon beta-1a in relapsing forms of MS (OPERA II; NCT01412333) (35). The data included in this study (clinical scores and imaging data) were from the baseline time point only. We also recruited 41 age-/gender-matched healthy control subjects (HC) (mean age ± SD = 34.9 ± 10.1; 14 male and 27 female) who were not part of the randomized study. Ethics approval was received from the university's Clinical Research Ethics Board; and all subjects provided written, informed consent. HC did not have psychiatric, medical, cognitive, or other conditions that caused an inability to participate in an MRI study. Patients had Expanded Disability Status Scale (EDSS) scores ranging between 0 and 4 (median EDSS = 2) (Table 1), indicating a mildly affected cohort.

**Table 1:** Demographics of MS patients.

	**HC subjects Mean ± SD**	**MS patients Mean ± SD**
Age	34.85 ± 10.1	37.20 ± 9.5
Gender	(14 male/27 female)	(10 male/15 female)
EDSS	ND	2.14 ± 0.95
Disease duration (months)	ND	67.08 ± 64.85
PASAT	ND	42.44 ± 15.38
SDMT	ND	49.88 ± 11.03
LCVA	ND	40.92 ± 9.65

*HC, healthy control; MS, multiple sclerosis; EDSS, Expanded Disability Status Scale; LCVA, low-contrast visual acuity; PASAT, Paced Auditory Serial Addition Test-3 s; SDMT, Symbol Digit Modalities Test; ND, no data*.

### Clinical Tests

All patients performed the MS functional composite (MSFC) battery (36) including the PASAT-3 s. They also performed a low-contrast visual acuity (LCVA) and the SDMT. As the purpose was to explore the potential functional biomarkers related to cognition, the sub-tests that primarily reflected ability of motor and physical coordination in MSFC, such as the Timed 25-Foot Walk and 9-Hole Peg Test, were not included in the study. Although the LCVA does not estimate cognitive function, this outcome was included in the analysis to explore whether clinically common visual problems were related to brain connectivity. The raw scores of these tests were used in the analyses in this study.

### Image Acquisition

Images were acquired with a Philips Achieva 3.0 Tesla MRI scanner (Best, The Netherlands). We collected 3D T1-weighted images with CLEAR homogeneity correction with 1 × 1 × 3 mm^3^ resolution. In detail, the 3D T1-weighted gradient echo scan was acquired with TR = 28 ms, TE = 4 ms, 60 axial slices acquired at 3-mm slice thickness, in-plane voxel size = 1 × 1 mm^2^, flip angle = 27°, and field of view (FOV) = 250 × 188 × 180 mm^3^. After structural image acquisition, 8 min of rs-fMRI data were acquired with an echo-planar imaging sequence with 3 × 3 × 3 mm^3^ resolution, 2,000-ms TR, 36 slices, 90° flip angle, and 240 volumes. Participants were instructed to close their eyes and to not think about anything in particular.

### Image Preprocessing

Several image preprocessing steps were applied to the fMRI data including slice-timing, isotropic reslicing, and motion correction in MATLAB using SPM8 functions (the Well-come Trust Center for Neuroimaging, UK) and in-house Matlab code. Subjects who showed more than 2° of rotation and 2 mm of translation were supposed to be removed from the analysis, but none of the subjects fulfilled the criteria. We further investigated the motions in patients; and the average displacements for translation and rotation are 0.32 ± 0.3 mm and 0.0051 ± 0.004°, respectively. FLIRT (the FMRIB Center, UK) was used to register fMRI images and structural images. T1-weighted images were used for cortical and subcortical parcellation, which was carried out using FreeSurfer software (version 4.5.0, Massachusetts General Hospital, USA) and parcellated based on the Desikan–Killiany atlas. After parcellation, 38 cognition-related regions of interest (ROIs) were chosen (Table 2). These ROIs, especially the ones in the frontoparietal areas and association cortex, have been commonly reported as important regions for cognitive function, especially higher-order function, in the literature and were chosen by an experienced neuropsychologist and a senior neurologist (37–43). These ROIs are located in the frontal (six ROIs), parietal (six ROIs), temporal (four ROIs), occipital (one ROI), and cingulate cortices (two ROIs). As the frontoparietal areas and association cortex were the main targets, subcortical regions were not included. The mean time courses over all voxels within one region in fMRI data were extracted from the given ROIs and detrended. All calculations were done in the subject's native space.

**Table 2:** The 38 ROIs (FreeSurfer version 4.5.0) in the study (19 bilateral regions).

**Bilateral ROIs**	
Frontal pole (front-pole)	Parietal and occipital junction areas (par-occi)
Superior frontal gyrus (front-sup)	Superior occipital gyrus (sup-occi)
Middle frontal gyrus (front-middle)	Anterior cingulate cortex (cACC)
Inferior prefrontal cortex (inf-prefrontal)	Posterior cingulate cortex (PCC)
Temporal pole, insula cortex, amygdala (temp-pole/ins/amyg)	Precuneus (precun)
Superior temporal cortex (sup-temp)	Medial orbitofrontal cortex (med-OFC)
Posterior parietal cortex (pos-par)	Lateral orbitofrontal cortex (lat-OFC)
Post central gyrus (postcentral)	Fusiform gyrus (fusiform)
Supramarginal gyrus (suprama)	Superior parietal cortex (sup-par)
Medial temporal lobe, hippocampus, parahippocampal gyrus (med-temp/hip/parahip)	

Several ROIs are grouped together to increase functional MRI signal-to-noise ratio.

*ROI, region of interest*.

### Methodological Considerations: Accurately Estimating Interhemispheric Connectivity

Technical issues may complicate interpretations of functional IC. For stationary interhemispheric connections, similarity between neural activities in homologous regions may not necessarily be based on transcallosal activity, but rather both hemispheres may be influenced by common brainstem and/or subcortical input. In other words, pairwise/Pearson's correlation, which is the most common approach to assess functional connectivity, estimates connectivity between two regions as well as regions that are potentially not of interest (44). This issue can be solved by partial correlation, whereby the relation between homologous pairs is assessed controlling for the activity in another region (44, 45). For dynamic connectivity, there is also the challenge of choosing the size of the sliding window when using this approach to estimate connectivity as it directly affects statistical power (46). The length of the window may affect connectivity results, and how to quantify connectivity changes across time so that the measures can be used to correlate with behavior is also challenging (47, 48). Calculating network features based on connectivity differences between time periods may show benefits for further analysis.

### Stationary Functional Interhemispheric Connectivity Analysis

We computed correlation coefficients to examine IC across homologous regions. For each subject, we first divided the time courses from *n* ROIs into *n*/2 homologous left (*L* = {left ROI1, left ROI2, …}), and right (*R* = {right ROI1, right ROI2, …}) pairs. For the *i*th pair (e.g., {*L*{*i*}, *R*{*i*})}, we examined the remainder right and left homologous pairs {*L*{1, …, *n*/2, ≠ *i*}, *R*{1, …, *n*/2, ≠ *i*}} and computed both the simple correlation (Pearson's *r*) and the partial correlation conditioned on both *L*{*i*} and *R*{*i*}. We then computed the sum of the *differences* in correlations and partial correlations between the remaining homologous pairs and attributed the result to {*L*{*i*}, *R*{*i*}}:

$$\begin{array}{l} D^{i}=\underset{j=1,\ldots ,\frac{n}{2},j\neq i}{\sum }|C\left(\left\{L\left\{j\right\},R\left\{j\right\}\right\}\right)\\ \;-C\left(\left\{L\left\{j\right\},R\left\{j\right\}\right\}|L\left\{i\right\},R\left\{i\right\}\right)| \end{array}$$

where *D*^*i*^ is the correlation difference attributed to the pair of ROIs *L*{*i*}, *R*{*i*}, *C*(^*^,^*^) is the correlation operator. The analysis was done in original fMRI time courses and the augmented time courses with one time point lag (details in Supplementary Material).

In order to determine if IC, described by *D*^*i*^'s, was associated with cognitive performance and whether certain homologous pairs had the largest influence on predicting clinical and cognitive scores, we performed regression with a sparsity penalty on the regression coefficients by implementing least absolute shrinkage and selection operator (LASSO) regression. Age was also included as a nuisance covariate. This algorithm attempts to find a balance between predicting cognitive scores and including as few interhemispheric connections as possible to make that prediction, which was one of the strategies to avoid overfitting.

To aid in interpretation, we then performed linear regression using the *fitlm* function in matlab and another linear regression with the connections selected by LASSO (Supplementary Figure 2 in the Supplementary Material). In order to assess significance of the results, we performed cross-validation. We permuted the rows of the selected connections and calculated the *p*-value of LASSO coefficients. This was repeated 100 times to create a null distribution, against which the unshuffled results were compared.

### Dynamic Functional Connectivity on Interhemispheric Connections

In order to estimate dFC, a sliding window approach, with window length (WL) of 30 time points (=60 s), was used; and it fulfilled the suggested criteria that WL should be ranged between 30 and 60 s (48). The window was shifted one time point forward in each simple correlation analysis, resulting in 211 windowed correlation matrices for each subject. Further features were obtained from these windowed correlation matrices. Flexibility of homologous connections FOCcs (flexibility of connectivity cross hemisphere in symmetric regions) was calculated using the connectivity differences of homologous connections between two adjacent windows. The values of connectivity differences were then summed up and divided by the total number of windows, which form one value representing how much the IC fluctuates across time. Specifically,

$$\begin{array}{l} FOCcs=\;\frac{1}{\left(L-1\right)}\;\sqrt{\overset{L}{\underset{t=2}{\sum }}\underset{i,j\;\in H}{\sum }{\left(M_{ij}\left(t\right)-M_{ij}\left(t-1\right)\right)}^{2}} \end{array}$$

where *H* is the set of *i, j* indices corresponding to all homologous connections, and *M*_*ij*_(*t*) represents *i, j*th element of the correlation matrix estimated at window *t*. *L* represents the total number of windowed correlation matrices (here, *L* = 211).

To explore the relations between dFC in IC, correlation analyses were carried out on FOCcs and cognitive scores.

## Results

### Processing Speed Performance

As the HC did not perform cognitive tests in the study, we compared our MS subjects' PASAT and SDMT performance with a previous study that included normative data in order to verify whether the patients showed cognitive decline (49). Our patients demonstrated mean SDMT score 1.25 SD below the normative data in the previous study (our MS: PASAT 42.44 ± 15.38 and SDMT 49.88 ± 11.03; normative data: PASAT 48.0 ± 10.7 and SDMT 61.9 ± 9.6), but the mean PASAT performance in our MS did not show a difference lower than 1 SD. Individually, six subjects demonstrated impairments on PASAT (showed score smaller than 1.5 SD of normative data), and nine subjects were impaired on SDMT test.

This indicated that most subjects had cognitive decline on both tests especially SDMT, but they did not reach the standard of impairments.

### Stationary Interhemispheric Connectivity

**Figure 1:**
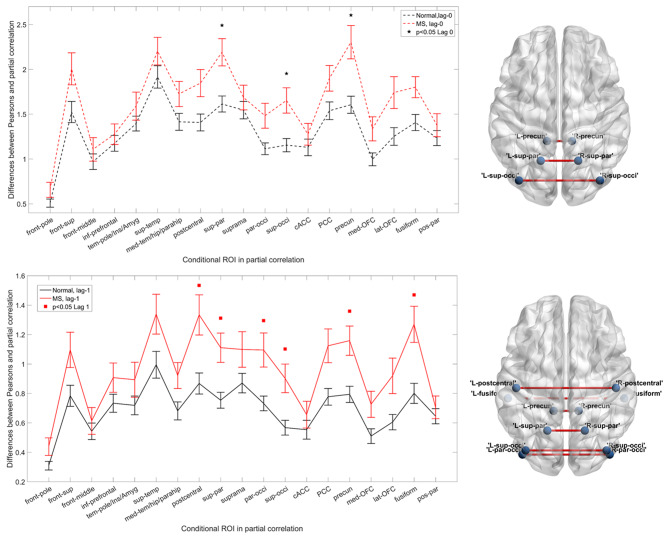
Functional interhemispheric connectivity in healthy controls (HCs) and multiple sclerosis (MS). Upper left panel and lower left panel represent instantaneous and delayed interhemispheric connectivity in MS (red) and HC (black), respectively. The differences between Pearson's correlation and partial correlation represent the relative importance (connectivity values) of the brain regions of interest [conditional region of interest (ROI)] when it is included or excluded from the analysis. A larger difference reflects greater importance of that region. Black stars and red squares indicate the connection pairs, which significantly distinguish the two groups in instantaneous and delayed connectivity [*p* < 0.05, false discovery rate (FDR) corrected], respectively. Upper right and lower right panels show the altered connections on a brain template. The visualization was done in the BrainNet Viewer (50). Abbreviations are shown in Table 2.

We observed significant differences between MS and HC (Figure 1). Figure 1 shows the differences between simple and partial correlation (*y* axis) across homologous regions (*x* axis) in the left panel. The changes in overall IC were significantly different [corrected *p* < 0.05, controlled for false discovery rate (FDR)] between MS and HC for three bilateral regions using partial correlation: the superior parietal cortex, superior occipital gyrus, and precuneus. In addition, the mean difference for all regions was higher for the MS compared with the HC group, regardless of what pairs were conditioned.

### Prediction of Cognitive Scores

**Figure 2:**
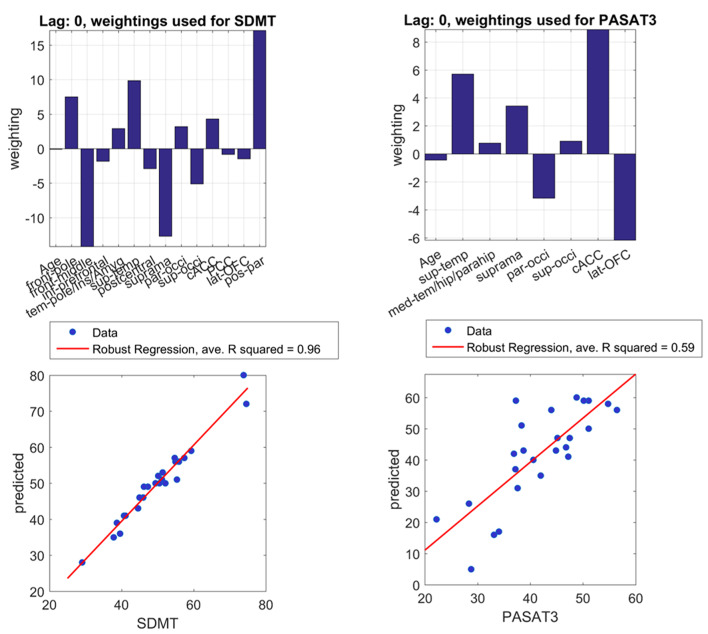
Important brain regions for cognitive performances. With least absolute shrinkage and selection operator (LASSO) regression, the upper left and upper right panels express weights of important connectivity pairs in predicting SDMT and PASAT, respectively. The lower panel shows positive correlations between real scores and predicted scores of SDMT and PASAT with the limited number of pairs. Real and predicted SDMT scores especially demonstrate a good correlation with average *R*^2^ = 0.96 in leave-one-out calculation. Predicted and real PASAT scores also show a strong correlation with average *R*^2^ = 0.59. Abbreviations are shown in Table 2. PASAT3, Paced Auditory Serial Addition Test-3 s; SDMT, Symbol Digit Modalities Test.

In linear regression, SDMT was significantly associated with IC pairs in the middle frontal, supramarginal, and posterior parietal regions (Table 3). In LASSO, even with the sparsity constraint on the regression coefficients, 13 and 7 ROI pairs were still included in the model to predict SDMT and PASAT scores, respectively. In Figure 2, the upper panel demonstrated the weightings of selected variables in LASSO. The variables with stronger weightings were influential to the model that was used to predict cognitive scores as shown in the lower panel. The signs of weightings represented how the variables influenced/contributed in the model in regard to the predicted variables. The variables with positive weightings tended to be positively associated with the response variable in the model, whereas negative weightings indicated negative associations. Age and several connectivity pairs jointly predicted SDMT scores (average *R*^2^ = 0.96, leave one out) including the frontal pole, middle frontal gyrus, inferior prefrontal cortex, temporal pole, insula, amygdala regions, superior temporal gyrus, postcentral gyrus, supramarginal gyrus, parietal and occipital junction areas, superior occipital lobe, anterior cingulate cortex, posterior parietal cortex, lateral orbitofrontal cortex, and posterior parietal gyrus. On the other hand, in addition to age, the superior temporal gyrus, medial temporal gyrus, hippocampal regions, supramarginal areas, parietal occipital regions, superior occipital gyrus, anterior cingulate cortex, and lateral orbitofrontal gyrus were highly associated with PASAT performance (average *R*^2^ = 0.59, leave one out). Interestingly, the three significant IC pairs that were associated with SDMT performance in the linear regression model could also predict SDMT in the LASSO regression.

**Table 3:** Estimated coefficients in a linear regression model with IC pairs and SDMT score.

	**Estimated Coefficients**			
	**Estimate**	**SE**	***t*-stat**	***p*-value**
(Intercept)	39.287	6.7822	5.7927	0.00216
x1	8.8104	6.425	1.3713	0.22863
x2	−3.2309	7.0069	−0.4611	0.66409
x3 mid-front	−16.867	3.939	−4.2819	**0.007849**
x4	−2.3876	5.2513	−0.45466	0.66841
x5	4.301	3.5032	1.2277	0.27419
x6	11.912	5.4428	2.1887	0.080232
x7	−2.1318	3.5704	−0.59709	0.57646
x8	−2.1629	3.2625	−0.66296	0.53669
x9	1.0862	4.5438	0.23906	0.82055
x10 supra	−16.596	5.5399	−2.9957	**0.03025**
x11	1.4426	6.4013	0.22535	0.83063
x12	−8.9211	5.5025	−1.6213	0.16588
x13	6.2903	4.2199	1.4906	0.19626
x14	−0.61528	8.2131	−0.07492	0.94319
x15	1.3835	8.5216	0.16235	0.87739
x16	0.46253	4.6265	0.099974	0.92425
x17	−1.3255	3.8491	−0.34438	0.74458
x18	3.4215	6.8526	0.49929	0.63876
x19 post-par	22.733	6.8725	3.3078	**0.021291**

Number of observations: 25, error degrees of freedom: 5.

Root mean squared error: 4.54.

R^2^: 0.965, adjusted R^2^ 0.831.

F-statistic vs. constant model: 7.2, p value = 0.019.

*mid-front, middle frontal gyrus; supra, supramarginal gyrus; post-par, posterior parietal cortex*.

*The bold values indicate the significance of connectivity pairs in the linear regression model*.

Finally, in the permutation test, *p* = 0.0009 for SDMT and 0.007 for PASAT against null distribution.

### Dynamic Aspect of Functional Interhemispheric Connectivity

**Figure 3:**
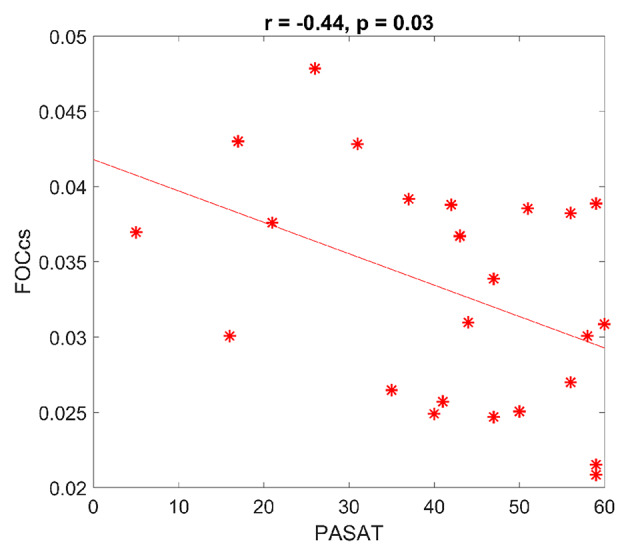
Dynamic functional connectivity and cognition in multiple sclerosis (MS). FOCcs, which represent dynamic interhemispheric connectivity, are negatively correlated with Paced Auditory Serial Addition Test (PASAT) scores. FOCcs: flexibility of homologous connections. Each * represents each dataset.

A sliding window approach was used, and a network measure was calculated to summarize the connectivity changes of interhemispheric connections (i.e., FOCcs). FOCcs showed a negative correlation with PASAT scores (*r* = −0.44, *p* = 0.03) (Figure 3), illustrating that better PASAT performance was associated with smaller flexibility of interhemispheric connections. However, this measure did not show differences between MS and HC groups. None of the other clinical or cognitive scores demonstrated correlation with FOCcs. Supplementary Material showed similar results with different parameters (different WL) using the same approach (Supplementary Figure 3).

## Discussion

### Enhanced Interhemispheric Connectivity May Be a Result of Adaptation and Compensation in Multiple Sclerosis

We found overall enhanced IC in MS subjects as demonstrated in Figure 1, which is consist with prior studies (51–54). In this study, we discovered significantly altered *functional* IC in MS through an approach that specifically assessed homologous connections. Previous fMRI studies have reported decreased functional connectivity in MS (51, 55), whereas increased functional connectivity has also been reported during rest as well as tasks (51–53), which may reflect compensatory mechanisms, results of adaptation, and/or loss of cognitive flexibility (54, 56, 57). For example, within the default mode network areas, several cortices demonstrate enhanced functional coupling in MS, which is associated with a concomitant loss of cognitive efficiency and may be a maladaptive process (56).

In this study, we propose a direct measure representing IC, which made the methodology in this study unique. Supplementary Figure 4 in the Supplementary Material describes the rationale and mathematical explanations of this approach in detail. Because the correlation differences (i.e., the actual IC values as acquired by subtracting direct correlation from full correlation) were consistently larger across all regions in MS (Figure 1) and the overall IC was also larger in the MS group, there is both enhancement and *homogenization* of IC in MS, meaning that the interhemispheric connections in MS are more likely to be affected by each other. If one connection is altered, the rest of the connections are more likely to be influenced as well. This also implies that interhemispheric connections in MS lose their ability to independently modulate neural communication between homologous regions. Instead, the homologous regions require further support from other connectivity pairs in order to transfer information across the two hemispheres, which indicates that enhanced interhemispheric communication could be an early compensatory change in MS.

However, recent research has questioned whether increased functional connectivity can simply represent compensation/adaptation in MS or whether the connectivity changes indicate a combination of both compensation and adaptation (58, 59). As our control subjects did not perform the same cognitive tests, it is hard to verify whether the enhanced IC solely represents compensatory effects or whether it can be an adaptive process. Regardless of this uncertainty, we propose that enhancement and homogenization of IC are a potential biomarker for MS.

### Homologous Regions Across Cortices and Dynamic Interhemispheric Connectivity Are Associated With Cognitive Performance

In addition to the finding of overall increased IC, we have also demonstrated that altered functional IC closely reflects performance on cognitive tests, namely, the SDMT and PASAT, with LASSO (Figure 2). If the LASSO regression did not find significant results, all the coefficients would be zero, and therefore, the regression cannot predict behavioral scores. In addition, with a permutation test, the *p*-value also indicated that the model of LASSO is significant. Interestingly, even when we used a sparsity constraint on the regression in LASSO, we still found several ROI pairs across cortices, which were required for accurate prediction of cognitive performance. Thus, both SDMT and PASAT performance appear to be strongly associated with widespread IC. In other words, working memory, sustained attention, and processing speed reflected in PASAT and SDMT are more distributed across cortices rather than localized (57, 60).

Nevertheless, some cortical regions showed different weighting to the cognitive performance. For SDMT, LASSO regression has shown that regions across cortices, especially the frontal pole, superior temporal gyrus, and posterior parietal cortex, demonstrated positive weighting, whereas the middle frontal and supramarginal gyrus presented stronger negative weighting to the performance in MS. Similarly, the regions shown with linear regression were all included in LASSO regression with the same sign of weighting (i.e., positive and negative). This linear combination of weighting indicated that even though different ROIs might show opposite effects to the performance, the coordination between these positive and negative effects might be the key to facilitate processing speed ability. In other words, some regions may show positive weighting of connectivity to *support* cognitive function, whereas other regions may show negative weighting of connectivity (i.e., suppress the model) to *modulate* performance. For PASAT, fewer ROIs with strong weighting were required to predict performance. The strong positive weighting from the superior temporal gyrus, supramarginal gyrus, and anterior cingulate cortex can predict and support sustained attention, working memory, and processing speed together with the negative weighting from the parietal occipital junction and lateral orbitofrontal gyrus to modulate performance in MS. Compared with SDMT, fewer ROIs were selected to predict PASAT performance. Because during the test, sustained attention was highly demanded as well as the skills with high cognitive load, the brain might purposely exclude non-crucial ROIs to save brain energy for the processes, which is especially important in the diseased brain. Overall, our results not only reinforce that 1) distributed regions support these cognitive performances (61–63) but also indicate that 2) the coordination between positive and negative effects of IC may be the key to facilitating cognitive performance. Although it is not clear how different connectivity pairs modulate cognitive function exactly or why such patterns are crucial to cognition, the trade-offs in cognition with limited neural resource may provide insights (64, 65).

Although dynamic IC is not significantly different between MS and HC, we do observe relations between IC and processing speed performance, indicating that subtle differences of FOCcs are related to behavioral outcome in MS subjects. Such a relation was only observed with PASAT, implying that unique strategies may be needed in different tests. In other words, one test may require stronger and more static IC compared with the other test. As the subjects in this study are not severely impaired, this indicates that the impairments of dFC in early MS may not be manifest and that the measure cannot serve as a biomarker, but the subtle changes are sensitive to early alterations of processing speed and provide insights into how interhemispheric connections react to cognitive load.

### Contrasting Paced Auditory Serial Addition Test and Symbol Digit Modalities Test

Although both PASAT and SDMT are clinically used for investigating cognitive deficits in MS, numerous studies have reported the SDMT to be a more sensitive, valid, and reliable measure than the PASAT (18, 19, 49, 66–68). Indeed, we found a strong correlation between SDMT and IC measures (Figure 2, Table 3), consistent with prior studies suggesting that SDMT is more strongly associated with MRI measures (18, 69).

SDMT has been thought of as measuring mainly processing speed and selective attention over a short time frame (62), and in which other strategies related to complex processes (e.g., working memory) are not used compared with PASAT. Therefore, fewer factors can affect the measurement, and SDMT may better reflect impairments in processing speed. SDMT performance requires a great deal of long-range connections such as frontoparietal and frontal-occipital networks (62). In long-range connections, the existence of “hubs,” regions that show denser connections to other regions and play a central role in the network (70–72), improves efficiency of neuronal signal transfer to support brain function and facilitate cognitive processes (73, 74). Given that IC has been shown to be highly dependent on functional IC (75), we propose that processing speed ability may be strongly associated with hubs. As interhemispheric connections shape the centrality of regions (i.e., hubs) (75), performance of processing speed tasks should also be highly related to IC.

We also show that IC of the frontal pole, temporal pole, insula, amygdala, superior temporal cortex, parietal and occipital regions, anterior cingulate cortex, and posterior parietal cortex is important for SDMT performance (Figure 2), which is partially consistent with previous studies showing that parietal areas play a role in SDMT as well as frontal areas and occipital regions (61), which are considered to be long-range connections. Moreover, baseline connectivity in these regions can predict future processing speed (60), emphasizing the relationship between processing speed ability and connectivity in distributed cortices. The IC of regions such as temporal pole, insula, amygdala, and superior temporal cortex has not been mentioned in previous studies. Although we suspect that these regions act as mediators in information communication between frontal and parietal areas for a short time, there is not enough evidence to support the hypothesis, and how the IC of these regions mediates cognitive performance requires further research. For example, although the link between IC and hubs has been shown (75), whether the IC pairs are linked to each other through hubs or other regions ipsilaterally may require whole brain analysis.

In contrast, performance on PASAT may require information coordination between frontal, parietal regions, and cerebellum because activation patterns mainly locate within one hemisphere among these regions, which will not be captured by the IC measures examined here (61). However, we found that only PASAT scores were related to dFC and better performance is associated with lower dynamics (Figure 3). PASAT examines processing speed and working memory, which involves higher-order cognitive processes, and these processes have been proposed to be related to variation in functional connectivity (i.e., dFC) (26, 76). Note also that the performance of PASAT requires sustained attention, which coordinates the recruitment of task-relevant resource, the discharge of resources for task-irrelevant processes, and the selection of exhibition and inhibition of cognitive processes in healthy subjects (77, 78). Therefore, the neuronal mechanism of sustained attention should also be dynamic and flexible. However, our results indicate an opposite trend, whereby better processing speed, working memory, and sustained attention ability measured in PASAT is associated with lower variation of functional connectivity even though our subjects are not severely impaired. Perhaps such flexibility is dysfunctional in early MS even though the alteration is not statistically significant, and neither is the patient performance. The subtle changes of losing flexibility require stable neuronal resources to constantly main the function. Therefore, we propose that PASAT performance may require unfluctuating/rigid IC in MS, but further research is needed to draw firm conclusions including taking into account learning effects, anxiety, and patients burden while performing PASAT (79).

Taken together, our results imply that interhemispheric connections between temporal areas, anterior cingulate gyrus, and a portion of parietal regions are crucial in PASAT and that the connectivity inflexibility of these connections are associated with the test performance. Perhaps, the performance of PASAT requires more stationary connectivity to support focused attention and inhibit unnecessary information rather than constantly coordinating signals across time and brain areas in MS. These mechanisms may also reduce the connectivity cost during PASAT performance as presumably less fluctuating connections require less energy consumption, which helps accomplish such a demanding task in the diseased brain.

## Limitations

There are some limitations to our study. We emphasized ROIs associated with higher-order cognition especially in the association cortex and thus would not detect other types of connectivity disruption affecting the primary senses, such as vision. In addition, here, we aimed to study cortical connectivity so some subcortical regions, which might be important for high-order cognition, were not included. Therefore, our data were not sufficient to investigate cortico-striatal loops, for example. Moreover, although several processes were performed to ensure the data quality, we did not specifically investigate the motion effects and remove the potential noise source with the use of commonly applied methods such as RETROICOR or FIX toolboxes (80, 81). Owing to the lack of physiological data and limited number of subjects, we did not apply these methods. In the future studies, removing potential noise from fMRI data requires further investigation. Owing to the design of this clinical trial, we did not administer a comprehensive neuropsychological test battery to examine all cognitive domains, and some variables were not collected either such as education and socoeconomic status. Therefore, the results only represent the cognitive tests that have been commonly used in clinical trials. Nonetheless, the PASAT and SDMT are well-validated, sensitive, and widely used measures of cognition in MS; and inclusion of these measures allows our study to be comparable with previous imaging and cognition studies in MS. As education was not included as a nuisance covariance, we cannot estimate whether education modulates the cognitive performance in the current study. Future studies should seek to determine IC across a wide range of cognitive domains with the control of nuisance covariance. Moreover, task-driven fMRI was not included in the original study design. Because we propose a relation between IC and cognitive performance in this study, implementing task-driven fMRI may provide further insights for future research.

Although we made attempts to prevent overfitting, including cross-validation, more subjects would enhance the robustness of our results. Furthermore, because we only had cognitive scores for the MS group, we lack the knowledge of how SDMT and PASAT relate to IC in HCs. In addition, the patients were not recruited based on cognitive impairment, so our results might not be representative of MS cohorts with severe cognitive problems. Furthermore, even though the suggested criteria for the sliding window approach have been proposed, this approach is still under debate (46). Further research of dynamic connectivity with different approaches is needed to fully understand how network dynamics are related to clinical data. Finally, linking lesion burden and cognitive impairments has always been an interest in this field. As we also acquired structural MRI data, it would be important to explore the relations between structural damages, functional disruptions, and cognitive/clinical impairments in MS (Supplementary Material includes preliminary results) as recent studies have investigated whether structural or functional impairments can better predict processing speed performance (82). However, the focus of this study is the functional aspect of IC and its relation to cognitive performance. Linking structural changes, functional alterations, and behavior is beyond the scope of this study.

## Conclusion

This study found increased stationary functional IC in MS, which were related to worsened cognitive performance. We have demonstrated altered IC in MS: these connections are more homogeneous in MS as well as enhanced, which might possibly serve as biomarkers. Furthermore, SDMT and PASAT scores were robustly correlated with stationary IC. Moreover, we discovered that PASAT performance is negatively correlated with dynamic IC in MS, possibly demonstrating that sustained attention and high cognitive load are related to inflexibility of interhemispheric connection. The results provide insights into how interhemispheric connections are affected in MS and how they are related to cognitive performance, which enhances our understanding of the disease.

## Data Availability Statement

The datasets generated for this study are available on request to the corresponding author.

## Ethics Statement

The studies involving human participants were reviewed and approved by Office of Research Ethics, University of British Columbia. The patients/participants provided their written informed consent to participate in this study.

## Author Contributions

S-JL performed the analysis, interpreted the results, and wrote the manuscript. SK edited the manuscript, contributed to the study design, and coordinated the clinical trial. AL contributed to technical aspects. KM edited the manuscript and helped with interpretations. IV edited the manuscript and helped with study coordination. ZW supervised technical aspects. AT supervised the clinical trial. MM initiated the analysis, edited the manuscript, and supervised this study.

## Conflict of Interest

SK has consulted for Genzyme and received research support from Roche and Genzyme. AT has the following competing financial interests: Research funding from Chugai, Roche, Novartis, Genzyme, Biogen. Consultancy honoraria from Genzyme, Roche, Teva, Biogen, Serono. MM is supported by the UBC/PPRI Chair in Parkinson's Research. The remaining authors declare that the research was conducted in the absence of any commercial or financial relationships that could be construed as a potential conflict of interest.
