# Supplementary material for: Both Stationary and Dynamic Functional Interhemispheric Connectivity Are Strongly Associated With Performance on Cognitive Tests in Multiple Sclerosis
Source: Front Neurol. 2020 Jun 4;11:407. doi: 10.3389/fneur.2020.00407 (PMC7287147; doi:10.3389/fneur.2020.00407)
Supplement: Supplementary file 1 [file Data_Sheet_1.docx]

Supplementary Material

# Functional connectivity analysis: two sets of time course

In order to accurately estimate IC, we used a number of functional connectivity methods to ensure robust results. First, in addition to the ROI time course data sets, we created an augmented data set by including temporally and circularly shifted data 1 time lag for connectivity analyses (Figure S1). The motivation was to test whether we can predict the neuronal events at a given time point based on the event at a previous time point. Moreover, if the results derived from augmented data are conflicting to the results from instantaneous data, the methods may not be adequate. As the TR was 2 seconds, only 1 time lag was appropriate. If we shifted the data more than 1 time lag (i.e. more than 2 seconds), the time courses would not be able to reflect the real fMRI signals because the time lag would be longer than the sampling time. Therefore, in total we had four sets of time courses: instantaneous time courses of MS group (no lag), instantaneous time courses of HC group (no lag), delayed time courses of MS group (1 lag), and delayed time courses of HC group (1 lag). Each set of time courses contained information on 240 time points from n ROIs (i.e. 38 ROIs).

Figure S1 illustrates how we temporally and circularly shifted the data 1 time lag for connectivity analyses. We aimed to use the information from a previous time point to predict the information at the current time point, which is similar to the granger causality approach (1,2).


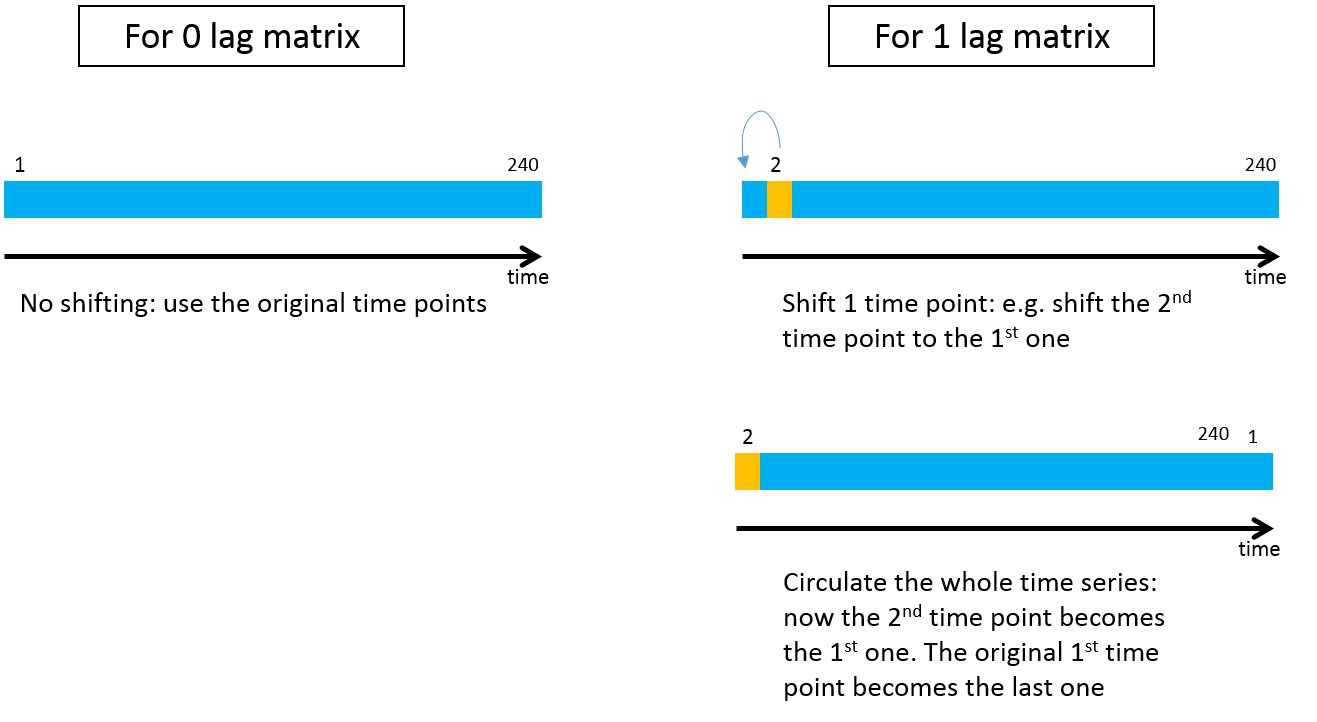


Supplementary Figure 1. Two sets of time series were included in the study. For instantaneous connectivity (0 lag), the original time series was used. For delayed connectivity (1 lag), we temporally and circularly shifted one time point as demonstrated.

## Regression model for connectivity and behavioural scores

Figure S2 demonstrates how the regression was carried out to investigate the relation between interhemispheric connectivity and behavioural performance. In the first calculation, we used a LASSO method to calculate β values based on interhemispheric connectivity values and raw scores. In the second calculation, we included the β values which have been calculated previously and interhemispheric connectivity values plus residual in a linear regression model to compute predicted scores. Finally, we plotted out raw and predicted scores as shown in figure 2.


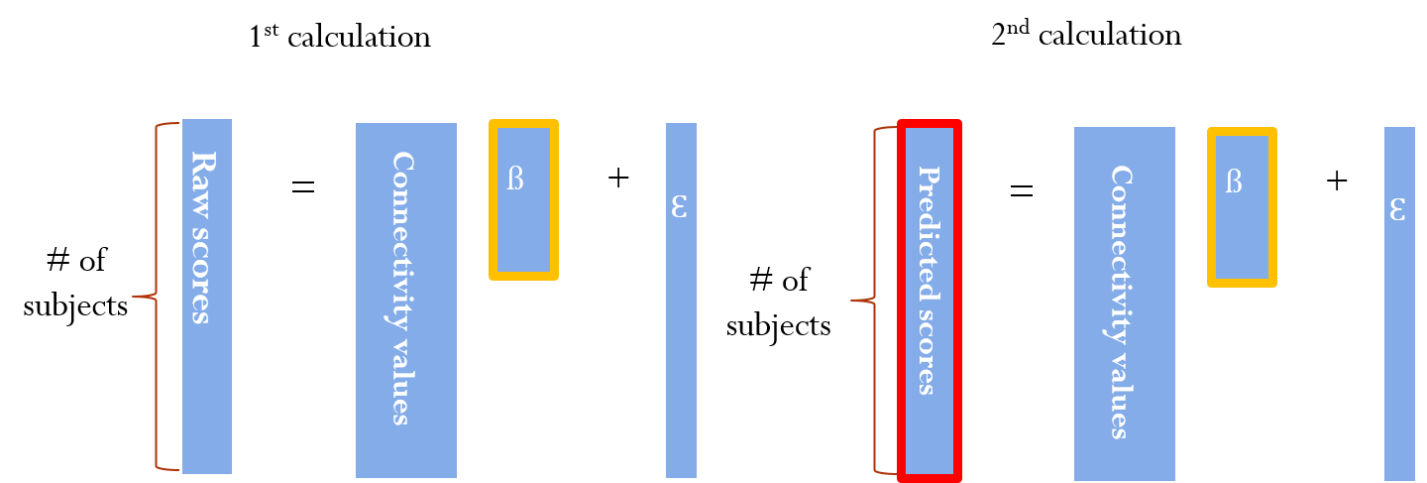


Supplementary Figure 2. The left panel shows the process of getting β values. The right panel describes the process of calculating predicted scores in a linear regression model.

## Results: dynamic functional connectivity on interhemispheric connections

One of the issues with the sliding window approach is the suspicion that different parameters (such as window length (WL)) may result in distinct findings. In our analysis, we tried different parameters with WL 30 (presented in the main article) and WL 40 time points (presented in the supporting information). Results were similar in terms of the correlation between network measures and PASAT scores. With WL 30, FOCcs (dynamic interhemispheric connectivity) was only correlated with PASAT with r = -0.44 and p = 0.03 (Figure 4). With WL 40, FOCcs only showed correlation to PASAT with r = -0.47 and p = 0.02 (Figure S3). Therefore, in our cohort, different WL did not cause distinct results, which also implies that the measure of dynamic interhemispheric connectivity is robust.


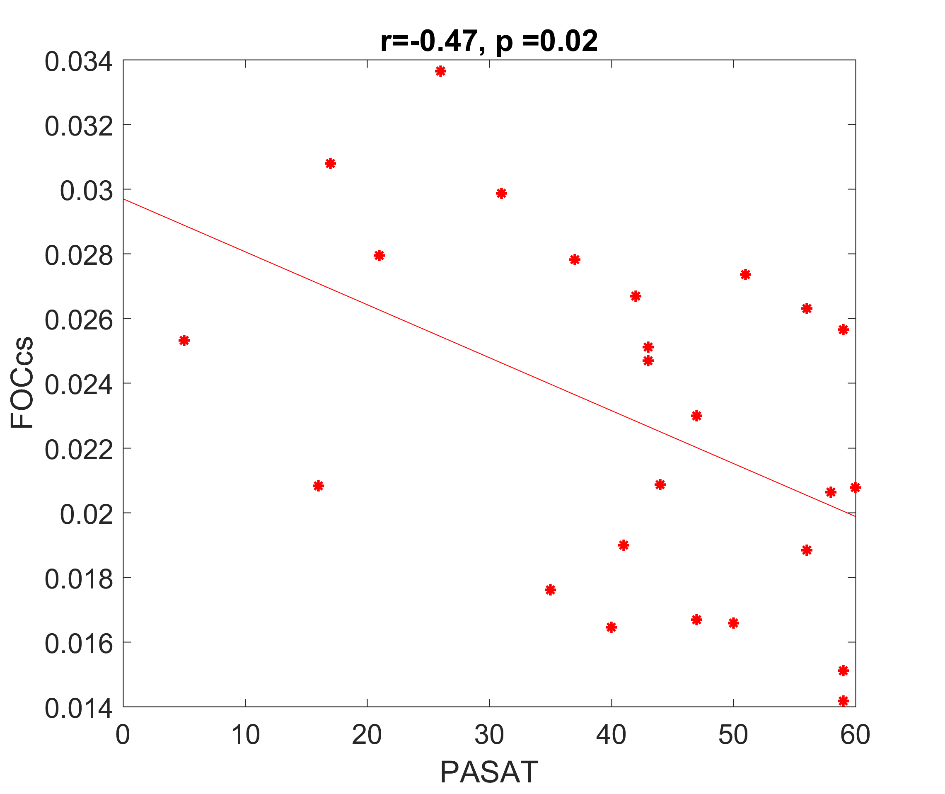


**Supplementary Figure 3.** The results with WL 40 in the sliding window approach.

# Discussion: partial correlation

Partial correlation can perhaps best be visualized in geometric fashion: for a given Pi(L,R) pair, where L and R are the same ROI in the left and right hemisphere and P is the correlation coefficient, we first projected the rest of the data pairs P1…n/2,≠i (L,R) onto the hyperplane that is perpendicular to Pi(L,R), before assessing the cosine of the angles between the P1…n/2,≠i (L,R) projections on the hyperplane. In effect, this assesses how “typical” the correlation between Pi(L,R) was to all P(L,R) pairs – if all P1…n/2,≠i (L,R) pairs interacted in the exact same way as Pi(L,R), the conditioning would have a large effect, and the difference between the Pearson’s r and partial correlation would be large. Note that by conditioning both on Pi(L,R) and Pi(L,R) that had been lagged by 1 time point, we are investigating how “typical” Pi(L,R) is compared to other ROIs and how robust it is over time (i.e. whether the connectivity lasts during the next time point).

Figure S4 explains partial correlation and how it conditions other variables in a geometric fashion. For a given P_i_(L,R) pair, we first projected the rest of the data pairs P_1…n/2,≠i_ (L,R) (shown as P_j_(L,R) in figure A4) onto the hyperplane (Si) which is perpendicular to P_i_(L,R), before assessing partial correlation, which is the cosine of the angles (φ), between the P_j_(L,R) projections on the hyperplane. In this case, we assessed partial correlation coefficient between P_j_(L,R) after controlling for the P_i_(L,R).


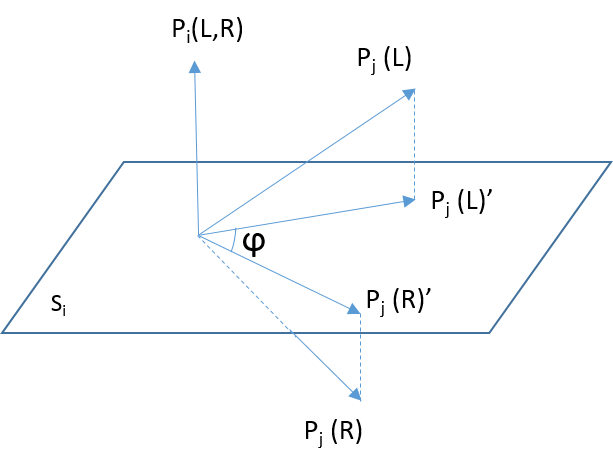


**Supplementary Figure 4.** The figure explains partial correlation using a geometrical demonstration in this study. P_i_(L,R) represents a given connectivity pair between left and right homologous regions which we need to condition; while P_j_(L,R) refers to the rest of the connectivity pairs for which we would like to assess partial correlation coefficients. This term was described as P_1…n/2,≠i_ (L,R) in the main article. P_j_(L,R)’ means the projections of P_j_(L,R).

# Limitation: Linking lesion burden, functional connectivity, and clinical data

Although the focus of the paper is not about linking structural damage, functional connectivity, and cognitive performance in MS, we did perform extra analyses to explore whether lesion burden is related to functional interhemispheric connectivity (IC) and/or cognitive performance in our cohort. First, we calculated total lesion volume to represent lesion burden (results shown in Supplementary table 1). We then performed correlation analysis on lesion volume and each pair of functional IC (i.e. one connection was included for each calculation) as reported in the main article. No significant results were observed. In another correlation analysis, we correlated lesion volume with the summation of all 19 IC pairs to form a value representing global IC. Instantaneous global IC did not show significant correlation. The delayed global IC was positively correlated with lesion volume (r=0.43, p=0.03) (Supplementary figure 5), but the results did not survive for multiple comparisons. In addition to associating structural damage and functional connectivity, we also carried out correlation analysis on lesion volume and cognitive performance. However, no significant correlations were revealed.

| Patient | Lesion volume mm^3^ |
| --- | --- |
| OPE_019 | 2869.16 |
| OPE_020 | 6204.13 |
| OPE_021 | 9207.60 |
| OPE_022 | 345.79 |
| OPE_023 | 334.35 |
| OPE_025 | 7701.58 |
| OPE_026 | 2543.38 |
| OPE_032 | 1506.02 |
| OPE_033 | 1000.21 |
| OPE_035 | 3909.37 |
| OPE_037 | 10156.37 |
| OPE_038 | 917.33 |
| OPE_039 | 214.33 |
| OPE_041 | 14031.45 |
| OPE_043 | 9407.64 |
| OPE_045 | 0 |
| OPE_046 | 7895.90 |
| OPE_051 | 2220.46 |
| OPE_052 | 1494.59 |
| OPE_053 | 2374.77 |
| OPE_058 | 6618.50 |
| OPE_059 | 2274.75 |
| OPE_060 | 1157.38 |
| OPE_061 | 1157.38 |
| OPE_062 | 1094.51 |
| mean±SD | 3865.48±3872.7 |

**Supplementary Table 1**. Lesion volume in MS subjects.


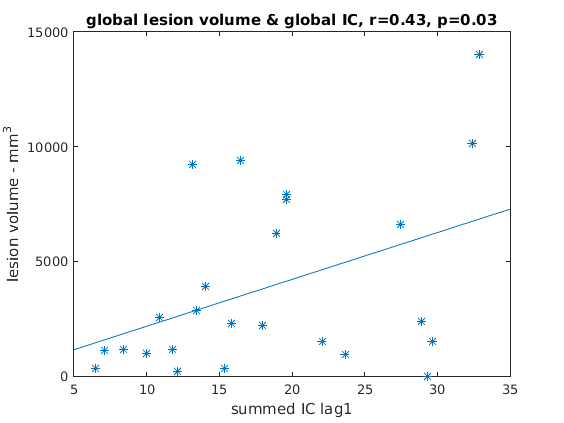


**Supplementary Figure 5.** Delayed global IC is correlated with lesion volume without multiple comparisons.

# References

1. Bressler SL, Seth AK. Wiener–Granger Causality: A well established methodology. *NeuroImage* (2011) **58**:323–329. doi:10.1016/j.neuroimage.2010.02.059

2. Granger CWJ. Investigating Causal Relations by Econometric Models and Cross-spectral Methods. *Econometrica* (1969) **37**:424–438. doi:10.2307/1912791
